# Elastic properties of leukemic cells linked to maturation stage and integrin activation

**DOI:** 10.1016/j.isci.2025.112150

**Published:** 2025-03-04

**Authors:** Ceri J. Richards, Albertus T.J. Wierenga, Annet Z. Brouwers-Vos, Emmanouil Kyrloglou, Laura S. Dillingh, Patty P.M.F.A. Mulder, Georgios Palasantzas, Jan Jacob Schuringa, Wouter H. Roos

**Affiliations:** 1 Moleculaire Biofysica, Zernike Instituut, Rijksuniversiteit Groningen, 9747 AG Groningen, the Netherlands; 2 Department of Experimental Hematology, University Medical Center Groningen, University of Groningen, 9713 GZ Groningen, the Netherlands; 3 Nanostructure Materials and Interfaces, Zernike Institute for Advanced Materials, University of Groningen, 9747 AG Groningen, the Netherlands; 4 Pharmaceutical Analysis, Groningen Research Institute of Pharmacy, University of Groningen, 9713 AV Groningen, the Netherlands

**Keywords:** Health sciences, Natural sciences, Biomechanics

## Abstract

Acute myeloid leukemia (AML) remains challenging to cure. In addition to mutations that alter cell functioning, biophysical properties are modulated by external cues. In particular, membrane proteins that interact with the bone marrow niche can induce cellular changes. Here, we develop an atomic force microscopy (AFM) approach to measure non-adherent AML cell mechanical properties. The Young’s modulus of the AML cell line, THP-1, increased in response to retronectin, whereas knock-out of the adhesion protein *ITGB1* resulted in no response to retronectin. Confocal microscopy revealed different actin cytoskeleton morphologies for wild-type and *ITGB1* knock-out cells exposed to retronectin. These results indicate that *ITGB1* mediates stimuli-induced cellular mechanoresponses through cytoskeletal changes. We next used AFM to investigate the elastic properties of primary AML cells and found that more committed cells had lower Young’s moduli than immature AMLs. Overall, this provides a platform for investigating the molecular mechanisms involved in leukemic cell mechanoresponse.

## Highlights

• Microwells loosely immobilize non-adherent cells for AFM indentation
• Leukemia cell elastic modulus increases upon retronectin binding to *ITGB1*
• Retronectin binding to *ITGB1* alters actin cytoskeletal network morphology
• Immature AML cells have a reduced elastic modulus compared to mature cells

## Graphical abstract

**Figure:**
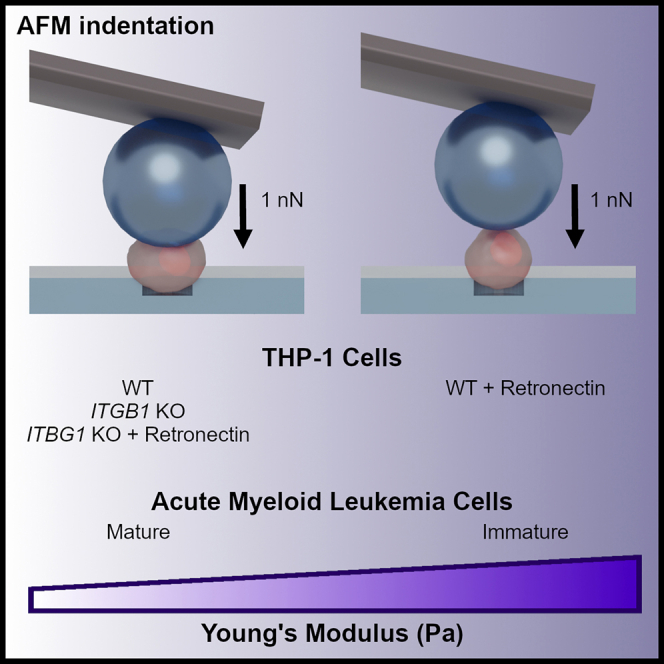

## Introduction

It is still difficult to cure acute myeloid leukemia (AML).^1^ In order to improve treatment options, a better understanding is needed on the molecular mechanisms that drive transformation and drug resistance.^2^ AML arises as a consequence of multiple somatic mutations in hematopoietic stem cells (HSCs) that accumulate upon age, resulting in impaired myeloid commitment and the accumulation of dysfunctional leukemic blasts in the bone marrow (BM).^3^^,^^4^ These mutations drive derailed signaling and cellular function, impacting on processes,` such as lineage commitment, immune evasion, and energy metabolism.^5^^,^^6^^,^^7^ Biophysical properties of cancer cells are also frequently altered,^8^ although little is known yet about mechanisms that drive such changes in AML, and how they impact on the transformation process.

Poor prognosis in AML is related to rare leukemic stem cells (LSCs) within the BM niche that are relatively drug resistant and are thought to give rise to relapsed disease.^4^^,^^9^^,^^10^ This BM niche is comprised of various cell types, including osteoblasts, osteoclasts, adipocytes, mesenchymal stromal cells, and various others.^11^^,^^12^ These interactions are critically important to control cell fate decisions such as maintenance of quiescence and self-renewal versus lineage specification.^11^^,^^13^^,^^14^^,^^15^^,^^16^ Also, they impact on drug sensitivities and resistance.^17^ In order to interact with the niche, HSCs and LSCs are equipped with an arsenal of plasma membrane proteins.^18^^,^^19^^,^^20^ These also include the family of integrins, which are essential for homing and adhesion characteristics of stem cells to the BM niche.^21^^,^^22^^,^^23^^,^^24^ Activation of integrins by extracellular matrix proteins such as fibronectin drives various intracellular processes, including activation of actin polymerization and biophysical properties of cells,^25^^,^^26^^,^^27^ and might therefore also impact on cellular elasticity. In this regard, when interacting with proteins present in the BM niche, differences in the expression of integrins on the plasma membrane of healthy and diseased stem cells may lead to differences in cell adhesion and elastic properties.^28^

Atomic force microscopy (AFM) has been previously used to measure the elastic properties of a range of cell types and tissues,^29^^,^^30^^,^^31^^,^^32^^,^^33^^,^^34^^,^^35^^,^^36^ with several AFM studies investigating elastic modulus specifically in the context of leukemia.^37^^,^^38^^,^^39^ AFM is a versatile technique that allows for measurements under physiological conditions, at piconewton and nanometer resolution, and with which one can measure various mechanical aspects (often simultaneously) such as force, adhesion, energy dissipation, and elasticity. Moreover there is a substantial theoretical and experimental framework for how to perform measurements and extract elastic modulus values,^40^^,^^41^^,^^42^^,^^43^^,^^44^ though this involves several intricacies and procedures must be carefully considered. The basics of such AFM measurement involve the indentation of the sample by a probe, after which the force-distance curve is fit to obtain the Young’s modulus value. However non-adherent cells, such as several leukemic cell lines, are easily pushed away by the probe during measurement. Previous studies have used coatings to loosely adhere non-adherent cells to a substrate thereby facilitating AFM measurements,^45^^,^^46^^,^^47^^,^^48^ however, this may also affect cell mechanical properties. Fewer works have managed to measure cells under non-adherent conditions with AFM by loosely constraining them in microwells or between micropillars,^39^^,^^44^^,^^49^ but measurements of such samples still remain a challenge.

In this study we have setup AFM protocols to quantify the elastic modulus (also called Young’s modulus) of primary LSC populations from AML patients. To do this, we developed micrometer-sized wells in order to constrain cells in space without adhering them to a surface. Our data indicate that the most immature CD34^+^/CD38^-^ LSCs display an increased Young’s modulus compared to more committed CD34^+^/CD38^+^ leukemic blasts, which in some cases can be further enhanced by integrin activation. Increased Young’s modulus values in response to retronectin in THP-1 leukemic cells is mediated via ITGB1 and is associated with actin cytoskeleton reorganizations.

## Results

### Measurements to determine the Young’s modulus of non-adherent cells

**Figure 1:**
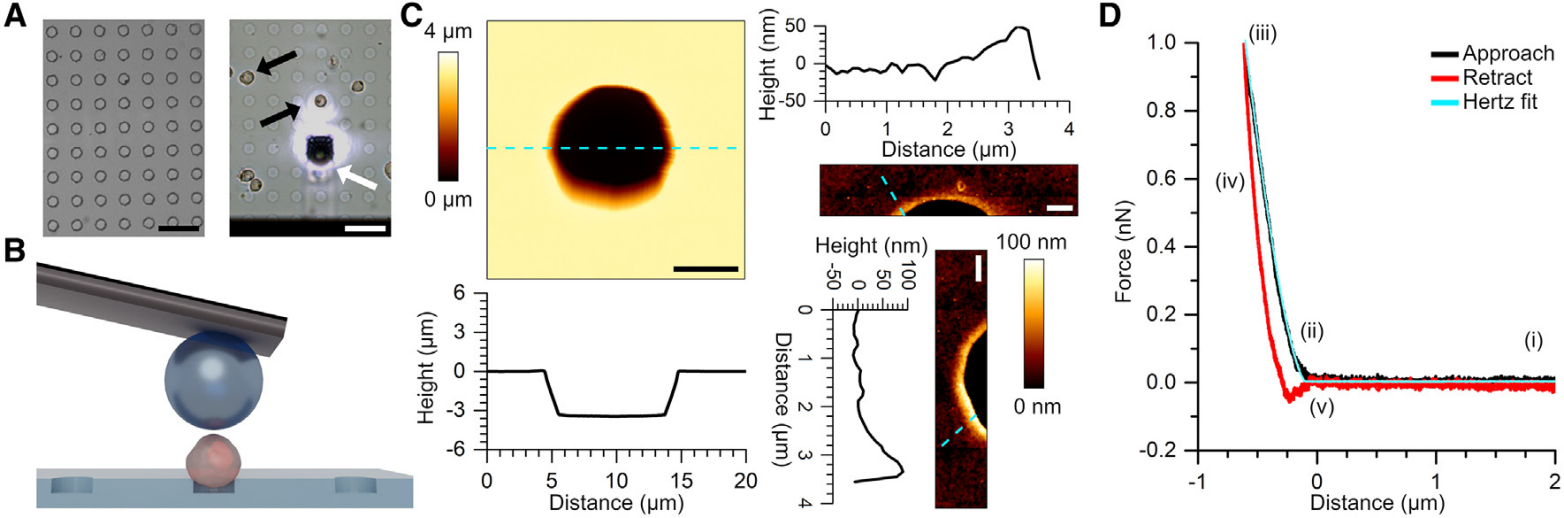
AFM approach to measure the Young’s modulus of non-adherent cells (A) Optical microscopy images of PFPE wells in air (left) and under experimental conditions (right) i.e., in liquid with cells deposited on the surface and the AFM cantilever present. The black arrows indicate two cells that have settled within the wells and are therefore suitable for measurement. The white arrow indicates the cantilever. The bright area in the center is the laser spot. Scale bars 40 μm. (B) Schematic representation of the experimental setup. The AFM cantilever has a 20 μm borosilicate sphere glued onto it. The sphere is placed centrally above a cell (red) that is constrained in a PFPE well. After which the probe is lowered onto the cell surface to perform a force-distance measurement. (C) AFM images of a PFPE well. The left of the panel shows an image of the whole well with the corresponding cross section (dashed line) shown in the graph below. The well is approximately circular with a diameter of ∼10 μm and depth of ∼3.5 μm. Note that there is some curvature at the bottom of the well but this is likely an artifact from imaging the deep structure with the AFM probe. Scale bar 5 μm. The right of the panel shows zoomed in AFM images of the well edges and the corresponding cross sections (dashed line) are shown in the graphs. A small lip of ∼50–100 nm is present around the edge of the well. Scale bars 2 μm. (D) Example force-distance curve of a THP-1 cell. The probe is approached (black line, i) and a force is registered once contact with the cell surface is made (ii). The probe continues to push on the cell up to a set force of 1 nN (iii), after which the probe is retracted (red line, iv) until it is no longer in contact with the cell (v). Some interaction between the probe and cell can be seen in the retract curve near point (v). The approach curve is fit with the Hertz model (cyan) to obtain the Young’s modulus of the cell.

We began by developing an approach to measure the Young’s modulus of non-adherent cells using AFM indentation. Inspired by previous work,^39^ perfluoropolyether (PFPE) substrates with a grid pattern of wells were fabricated^50^^,^^51^ (Figures 1A and 1B) in order to constrain suspension cells during the indentation process. AFM imaging confirmed a well diameter of ∼10 μm and depth of ∼3.5 μm (Figure 1C). Moreover a small ∼50–100 nm high lip around the well edge was observed, likely as a result of the fabrication process. Suspension cells were added to the substrate and left to settle. The cells which settled between the wells were readily pushed away by the AFM probe, while the cells that settled on the wells generally remained in position and were suitable for indentation (Figure 1A, arrows). Due to the well dimensions, only part of the cell volumes were located within the wells (Figure 1B), therefore the cells were not fully fixated and could still move within and out of the wells when sufficiently disturbed by either the AFM probe or external vibrations. Indentation measurements were performed with 20 μm borosilicate beads glued to AFM cantilevers (Figure 1B) in order to characterize the global cellular elastic properties.^52^

Similar to methodologies used in other reports,^44^^,^^47^ AFM measurements were performed by 15 repeat indentations over the center of each cell, resulting in 15 force-distance curves. Figure 1D shows an example of such a force-distance curve. Far from the cell surface, no force was recorded as shown in region (i) of the curve in Figure 1D. As the probe made contact with the cell surface, the probe deflected and a force was registered (ii), the probe continued to indent into the cell until a given set point of 1 nN (iii), after which the probe retracted (iv) until it was no longer in contact with the cell (v). To determine the elastic modulus of the cell, the region from point (i) to (iii) of the force distance curve was fit with the Hertz model (Figure 1D, cyan line).^41^ With a set point of 1 nN, typical indentation depths were 500 nm or more. In the context of leukemia, egress from the BM niche involves large cellular deformations through capillaries that can be as small as 3 μm.^53^ Though colloidal probe AFM indentation does not emulate the mechanism by which a cell is deformed through a capillary, investigation of the overall cellular elastic properties, i.e., including contributions from the plasma membrane, actin cortex, cell nucleus and microtubules, is of interest.^53^ Thus, the typical indentation depths used in this study not only probe the mechanical properties of the plasma membrane and actin cortex (typically within 200 nm of the plasma membrane) but also from other cellular structures, yielding the global cellular elastic properties.^54^

We noted changes in the elastic modulus of the cells across repeated indentations (Figure S1). The largest change in Young’s modulus occurred between the first and second indentation, which we attribute to the probe pushing the cell into a new position within the well. Further indentations led to both increases and decreases in elastic modulus values, but the magnitude of the changes were all reduced compared to the difference observed after the first indentation. Thus, we cannot rule out whether the changes in mechanical properties were caused by further repositioning of the cell within the well or whether it was a cellular response to the repeated indentations. Given the large difference between the first and second indentation and the moderate changes afterward, we chose to remove the first indentations from the datasets and used indentations 2–15 for subsequent analysis.

### Increased cell elastic modulus in response to retronectin is mediated via *ITGB1*

**Figure 2:**
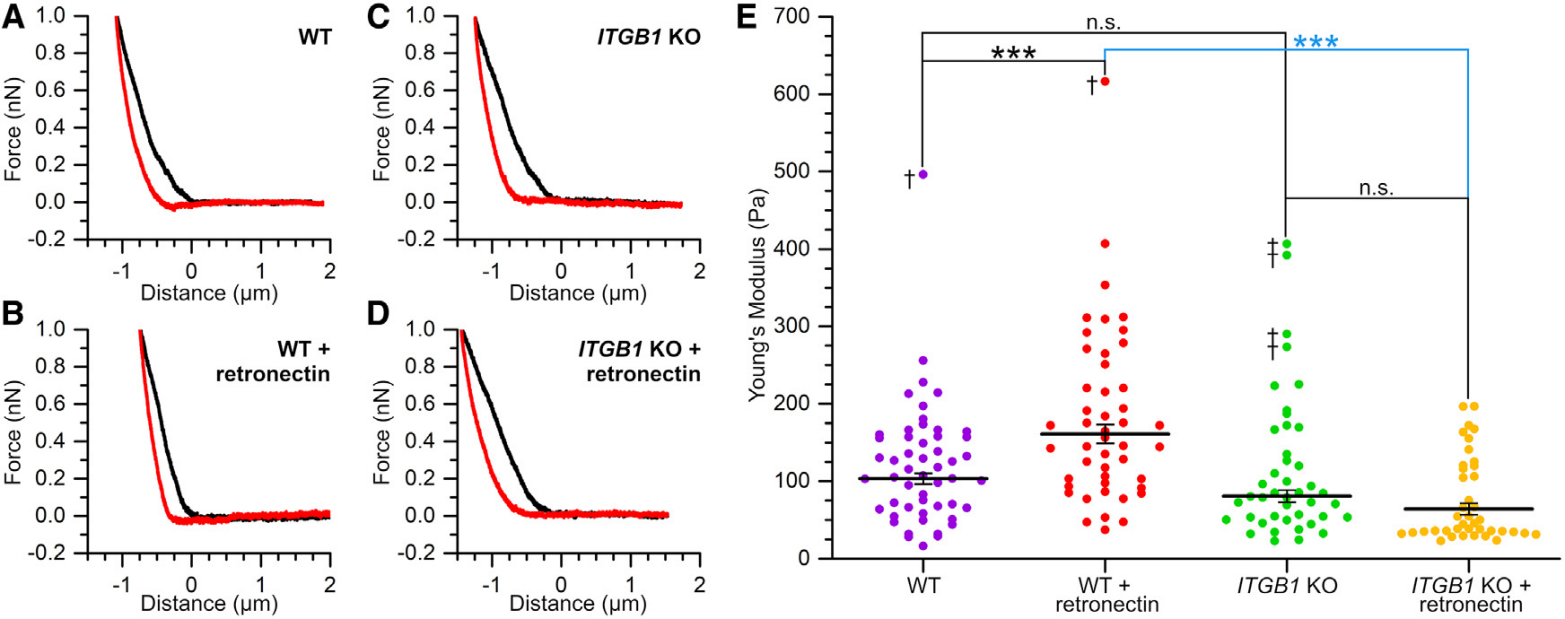
Elastic modulus measurements of THP-1 cells The Young’s modulus of THP-1 cells was measured using the AFM procedure outlined above. (A–D) Example force distance curves of wild type THP-1 cells (A) under control and (B) with retronectin added in solution, or *ITGB1* knock out THP-1 cells (C) under control and (D) with retronectin added in solution. Approach curves (red) and retract curves (black). (E) Distribution of the measured Young’s modulus values for cells under various conditions. Each point represents the average of 10–14 measurements performed on one cell. All experiments were performed in triplicate and outliers are indicated with the daggers. The horizontal bars show the distribution means and the error bars are the standard error across cells after outlier removal. The number of measured cells (after outlier removal) are 58, 46, 39, and 40 for WT, WT + retronectin, *ITGB1* KO, and *ITGB1* KO + retronectin, respectively. Significance testing was performed using a two sample t test with an alpha of 0.05. The following *p* values were obtained: *p* = 0.0004, 0.066, 0.180, and 4 × 10^−8^ for WT/WT + retronectin, WT/*ITGB1* KO, *ITGB1* KO/*ITGB1* KO + retronectin, and WT + retronectin/*ITGB1* KO + retronectin, respectively.

THP-1 cells were used as a model acute myeloid leukemia (AML) cell line. CRISPR-Cas9-mediated knock-out of the integrin *ITGB1* was performed by electroporation with Cas9 protein and gRNAs,^55^ and after flow sorting of ITGB1^-^ cells 98% of cells tested negative for ITGB1 (Figure S2). Adhesion experiments (Figure S3) confirmed that knock-out of *ITGB1* significantly reduced THP-1 adhesion to retronectin-coated plates. Wild type (WT) and *ITGB1* knock-out (*ITGB1* KO) cells were separately measured using AFM indentation under control conditions (in 50% cell culture medium and 50% phosphate buffered saline at 37°C). Figures 2A and 2C show example force-distance curves from the two cell types. The WT cells had an average Young’s Modulus of 113 ± 8 Pa (mean and standard error), and the *ITGB1* KO cells had a Young’s Modulus of 91 ± 9 Pa (Figure 2E and Table 1). There was no significant difference between the mean Young’s Modulus of the two distributions. Likewise, the proliferation rates were similar (Figure S4). Lastly, though cell area was found to have little influence on the measured elastic modulus (Figure S5), we also confirmed that the average cell areas of WT and *ITGB1* KO were similar (Table 1). Overall, the *ITGB1* KO did not appear to alter cell attributes (elastic modulus, area, and proliferation), therefore we could readily compare the effect of extracellular stimuli on WT and *ITGB1* KO cells.

**Table 1:** Elastic modulus measurements of THP-1 cells

Sample	*n*	Young’s Modulus (Pa)	Cell Area (μm^2^)
WT	51	113 ± 8	129 ± 24
WT + retronectin	46	171 ± 13	142 ± 30
*ITGB1* KO	39	91 ± 9	141 ± 24
*ITGB1* KO + retronectin	40	74 ± 9	123 ± 19
PFPE		19400 ± 600	

Average Young’s modulus values of THP-1 cells. Errors are the standard errors of the mean and *n* is the number of cells measured. Experiments were performed in triplicate. Cell areas were determined from optical microscopy and the values stated are averages and standard deviations. Measurements were also performed on the substrate. We note that due to the soft cantilever used we measure an amalgamation of the cantilever and substrate properties. However, this highlights that indentations on the substrate were readily distinguishable from indentations on cells.

We next measured the effect of retronectin on the elastic modulus of WT and *ITGB1* KO cells (Figures 2B and 2D). Retronectin was used as a model for the extracellular cellular matrix protein, fibronectin, which binds to the extracellular component of integrin dimers and modulates cellular responses.^27^ Though the addition of retronectin in solution may form a coating on the PFPE substrate, promoting the adhesion of the cells to the surface, we did not observe obvious signs of any such effect. The cells did not spread on the surface and were still readily dislodged from the wells with small disturbances, thus exhibiting similar behavior to the cells in the absence of retronectin.

Upon AFM indentation, we found that the Young’s modulus of WT cells incubated with retronectin was significantly increased compared to WT cells under control conditions (Figure 2E and Table 1). In contrast, the Young’s modulus of *ITGB1* KO cells exposed to retronectin did not show a significant difference compared to the same cells under control conditions (Figure 2E and Table 1). Accordingly, WT cells exposed to retronectin had significantly higher Young’s moduli compared to *ITGB1* KO cells exposed to retronectin.

To verify whether this result was affected by our analysis choice (to remove the first indentation) we also analyzed only the first indentation curves for each cell (Figure S6), where changes to the elastic properties induced by mechanical stimulation are minimal. We likewise found that knock out of *ITGB1* lead to no significant changes in the Young’s modulus upon exposure to retronectin compared to control conditions, whereas WT cells did show a significant increase. Therefore, *ITGB1* appears to have a significant role in the response of THP-1 cells to extracellular stimuli.

### ITGB1 modulates the actin network in response to retronectin

**Figure 3:**
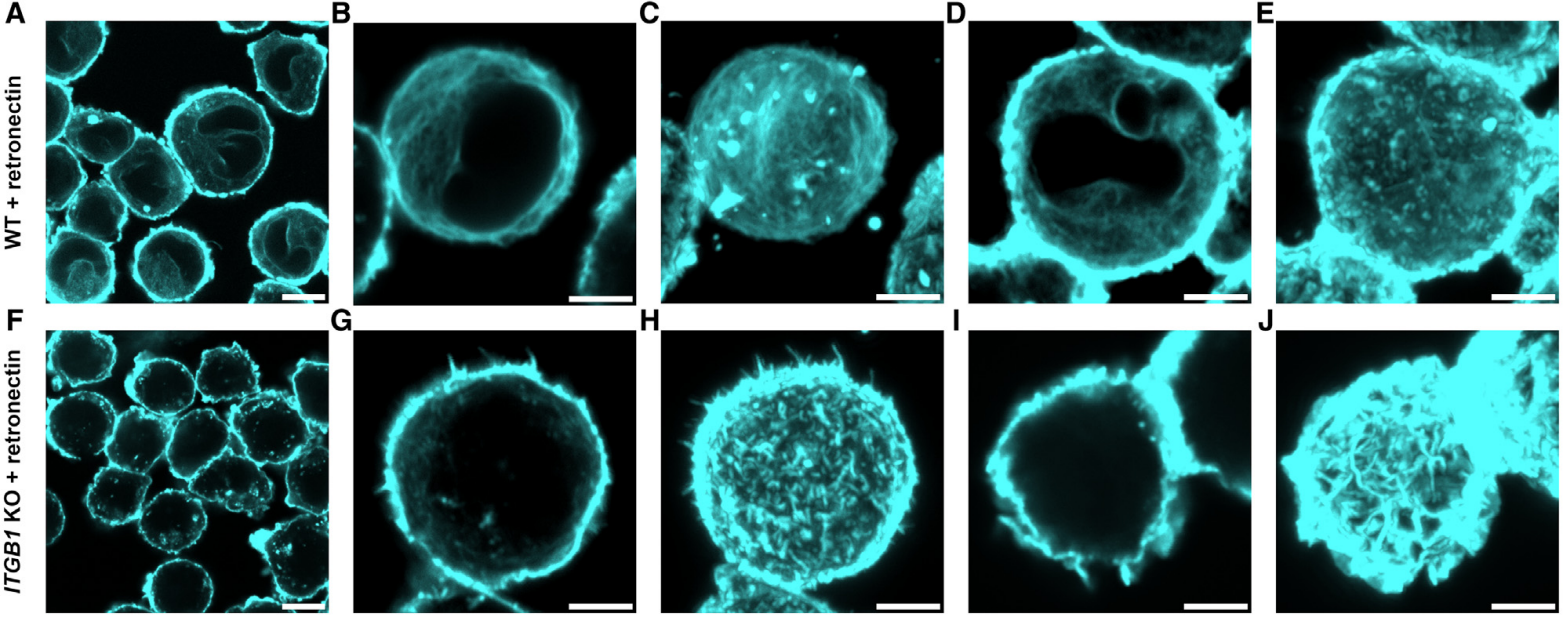
Actin staining of THP-1 cells exposed to retronectin THP-1 cells were seeded on retronectin coated slides and then exposed to additional retronectin in solution. Actin filaments were stained using phalloidin (cyan) and imaged with confocal microscopy. Experiments were performed in triplicate and multiple overview as well as high resolution images were obtained per repeat. Images of (A–E) wild type THP-1 cells, and (F–J) *ITGB1* knock out THP-1 cells. (A) Overview image of wild type cells. Actin staining can be seen at the membrane as well as at a lower intensity throughout the cell cytosol. (B–E) Higher resolution volumetric imaging of individual cells where (B and D) show an individual *z* slice of each cell, and (C and E) show the corresponding maximum z intensity projection of the entire cell volumes. An extended network of long actin filaments can be seen within the cell volumes. (F) Overview image of *ITGB1* knock out cells. Actin staining is mainly seen at the membrane as well as a few puncta within the cell cytosol. (G–J) Volumetric imaging where (G and I) show an individual *z* slice, and (H and J) show the corresponding maximum z intensity projections. Actin filaments within the cell cytosol appear as either short fragments or puncta, but in general there is little evidence of an extended network. Instead, there are many actin-rich structures extending from the cell membrane. Scale bars are 10 μm for panels (A and F) and 5 μm for panels (B–E, G–J).

Integrins bind to the actin cytoskeleton, which, in addition to the cell nucleus, is a major contributor to the Young’s modulus values measured by AFM approaches.^31^^,^^56^ Moreover, the organization of the actin network has been related to cellular mechanical properties.^32^^,^^33^^,^^37^ Therefore, we used confocal microscopy to assess the morphology of the filamentous actin (F-actin) network in THP-1 WT and *ITGB1* KO cells exposed to retronectin. Overview images of phalloidin stained cells already showed a clear difference in the distribution of F-actin in WT and *ITGB1* KO cells (Figures 3A and 3F, respectively, as well as Figure S7). WT cells exposed to retronectin showed high intensity staining at the cell membrane, as well as lower intensity staining throughout the cell cytosol. Single cell volume imaging (Figures 3B–3E) revealed long actin fibers throughout the cell volume. Contrastingly, overview images of *ITGB1* KO cells exposed to retronectin showed little cytosolic staining aside from several puncta, and most F-actin staining was localized at the cell membrane (Figure 3F). Higher resolution imaging (Figures 3G–3J) likewise showed that cytosolic actin staining was mainly in the form of puncta or short fibers. However, actin-rich plasma membrane projections are clearly visible in the three dimensional projections (Figures 3H and 3J) which are substantially less present for WT cells (Figures 1C and 1E).

In order to substantiate this qualitative conclusion, we also performed a quantitative analysis. The actin structures were identified within singular optical sections of the cells (taken at the midpoint of the cell) and were skeletonized to obtain the structure lengths, similarly to previous work.^37^ We note that the cortical actin was removed from the analysis as this typically formed one or two long skeletons that denoted the perimeter of the cell due to the high intensity and density of actin staining in this region. WT cells exposed to retronectin had a total actin network length per cell of 48 ± 7 μm and average structure length of 1.8 ± 0.2 μm, whereas these values were, 7 ± 1 μm and 0.86 ± 0.08 μm, respectively, for the *ITGB1* KO cells exposed to retronectin. Moreover, the procedure included background subtraction which removed much of the low intensity staining present within the cytosol of the WT cells, which was present to a much lesser degree for the *ITGB1* KO cells. This staining may be out of focus structures, or filaments that we were unable to sufficiently resolve with confocal microscopy. In the latter case, this would suggest our value for the total actin length per cell for WT cells is in fact an underestimate. Overall, the quantitative analysis shows that WT cells exposed to retronectin had a more extensive actin network consisting of longer structures compared to *ITGB1* KO cells exposed to retronectin.

We do note that under these conditions the cells were adhered to the surface and this may influence the cytoskeleton organization. In particular, WT cells adhere to the glass substrate whereas adhesion is reduced for the *ITGB1* KO cells. However, the cells maintained their spherical morphology for both WT and *ITGB1* KO cells, leading to only a minor fraction of the cell surface area being in contact with the substrate. Moreover, the staining patterns shown were also characteristic for images taken at the tops of the cells, where we anticipate surface interactions to have little effect on the cytoskeletal morphology. Thus we suggest that any surface adhesion effects plays a minor role in the actin morphology and measured stiffening response of the WT and *ITGB1* KO cells. Overall, our results show that retronectin binding to *ITGB1* induces alterations in the actin network distribution which co-occurs with increased THP-1 cell elastic modulus.

### Measurements of the elastic modulus of patient AML samples

We next studied the response of primary patient samples to extracellular retronectin. Patient-derived AML cells were sorted into the cell fraction that was positive for CD45 and the stem/progenitor cell marker CD34. These cells were further sorted into mature CD34^+^/CD38^+^ and immature CD34^+^/CD38^-^ LSC fractions (Figure S8 and Table S1 for population numbers). AML cells from three different patients that showed similarities in genetic mutations in terms of NPM1 and FLT3 were used (Table S2). AFM indentation was separately performed on the CD34^+^/CD38^+^ and CD34^+^/CD38^-^ AML cells from the three patients under control conditions, as well as in the presence of retronectin in the extracellular medium.

The AMLs derived from the three different patients showed different Young’s modulus values, for example the cells of patient 2 generally had higher elastic moduli compared to patients 1 and 3 (Table 2). Therefore, the samples from each patient were analyzed separately as opposed to the data being pooled across all patients. We also compared the average cell areas for each patient and condition and found that they differed to some degree but were generally within the error of one another (Table S3). Moreover, the differences in cell area did not generally appear to reflect the difference in Young’s modulus values.

**Table 2:** Measurements of the Young’s modulus of patient samples

Sample	Patient 1		Patient 2		Patient 3	
	*n*	Young’s Modulus (Pa)	*n*	Young’s Modulus (Pa)	*n*	Young’s Modulus (Pa)
CD34^+^/CD38^+^	22	32 ± 2	26	65 ± 10	22	29 ± 1
CD34^+^/CD38^+^ + retronectin	19	27 ± 2	26	64 ± 8	23	28 ± 1
CD34^+^/CD38^-^	10	53 ± 10	28	116 ± 10	28	43 ± 4
CD34^+^/CD38^-^ + retronectin	11	96 ± 20	24	85 ± 14	23	30 ± 1

Average Young’s modulus values for patient AML cells. Errors are the standard errors of the mean. *n* is the number of cells measured.

**Figure 4:**
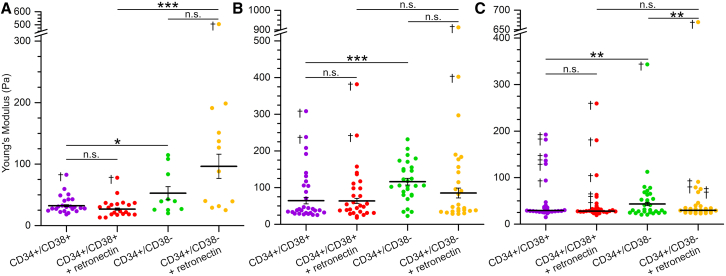
Stiffness measurements of patient AML samples Patient samples were sorted into CD34^+^/CD38^+^ and CD34^+^/CD38^-^ populations and measured using the AFM procedure outlined above. Distributions of the measured Young’s modulus values of cells from (A) patient 1, (B) patient 2, and (C) patient 3. Each point represents the average of 10–14 measurements performed on one cell. Outliers are indicated with the daggers. The horizontal bars show the distribution means and the error bars are the standard error across cells after outlier removal. For all patients, CD34^+^/CD38^-^ cells are significantly stiffer than CD34^+^/CD38^+^ cells. The addition of retronectin has no effect on CD34^+^/CD38^+^ cell stiffness for any of the patients, whereas it has mixed effects on the CD34^+^/CD38^-^ cells. The number of measured cells for each sample, *n*, is reported in Table 2. Significance testing was performed using a two sample t test with an alpha of 0.05. The *p* values are given in Table S4.

Figure 4 shows the populations of measured cell Young’s moduli for each patient and condition, while the population averages and errors are reported in Table 2. For each patient, the CD34^+^/CD38^-^ AML cells were found to have a significantly higher elastic modulus compared to the CD34^+^/CD38^+^ AML cells. Moreover, retronectin exposure did not significantly alter the Young’s modulus of committed CD34^+^/CD38^+^ cells for any of the patient derived samples. However, retronectin exposure had mixed effects on the elastic modulus of immature CD34^+^/CD38^-^ AML cells. A trend was seen for the AMLs of patient 1 where retronectin further enhanced the elastic modulus, but this did not reach significance. For the other patient AMLs retronectin did not induce an increase, and even resulted in a decrease in the Young’s modulus for patient 3.

We note that several cells were found to be outliers with much higher Young’s modulus values compared to the rest of the cell populations (Figure 4, daggers), in particular for patient 3. It is possible that these cells may represent less viable cells, or that they are from a sub-population of AML cells, which are known to be very heterogeneous. Nevertheless, using a less stringent method to detect outliers did not affect the overall outcomes of the significance testing, except the difference between retronectin exposed CD34^+^/CD38^+^ and CD34^+^/CD38^-^ AML cells became non-significant for patient 3. It would be very interesting to determine if these outliers do in fact originate from cellular sub-populations and consequently understand the biological origin and relevance of differing cellular mechanical properties. However, to sufficiently capture sub-populations, and consequently the effect of retronectin on the elastic modulus of these sub-populations, substantially more data would be required. In summary, we observed that the most immature LSC populations display higher Young’s moduli compared to more committed CD34^+^/CD38^+^ cell populations.

## Discussion

One of the most urgent challenges in the field of AML treatment is the frequent recurrence of relapsed disease, which is thought to be caused by therapy resistant quiescent LSC populations that escape treatment. Therefore, a thorough further understanding of underlying mechanisms that drive LSCs is warranted. It is increasingly becoming clear that biophysical properties also have a major impact on stem cell fate, including their sensitivity to drugs, but little has been revealed yet about their role in AML cells. To begin to address this, characterization of differences in the mechanical properties of the quiescent LSC population and more committed population is therefore of interest. Here, using optimized AFM measurements, we show that the most immature CD34^+^/CD38^-^ LSC populations display an increased elastic modulus as compared to more committed CD34^+^/CD38^+^ populations.

It was recently shown that in the normal hematopoietic system the most immature murine HSC populations have higher elastic moduli compared to more mature cells.^45^ This is controlled by the protein tyrosine phosphatase Ptpn21, as *Ptpn21*-deficient HSCs displayed decreased Young’s modulus and increased physical deformability and mobility.^45^ This was linked to hyper-phosphorylation of residue Tyr246 of the cytoskeleton-associated Septin1, a downstream target of Ptpn21.^45^ Little is known about the role of Ptpn21 in AML. It might be overexpressed in a subset of cases,^57^ but it is not frequently found to be mutated. One study, making use of the same *Ptpn21*^−/−^ mice, showed that leukemic transformation induced by the oncogene *MLL-AF9* developed more aggressively in the absence of Ptpn21.^38^ This was initially surprising to us since loss of Ptpn21 results in spontaneous mobilization, HSC defects and impaired hematopoiesis,^45^ so one might hypothesize that maintenance of LSC self-renewal would also require Ptpn21. The retroviral MLL-AF9 overexpression transplantation model might, however, differ from leukemia progression in patients. The authors proposed that the more aggressive leukemia development might not be directly related to alterations in signaling, but was attributed by the authors to cell intrinsic mechanical changes such as decreased tension and increased deformability. Possibly, this might relate to an easier egress of leukemic cells from the BM niche resulting in a higher leukemia burden in the periphery, in analogy with what has been suggested for metastasizing solid cancer cells,^29^^,^^58^^,^^59^ but further studies are needed to provide mechanistic insights.

In a previous report, AFM was also used to measure the Young’s modulus of leukemic cell lines. Clear differences were observed between the AML cell line HL60, the T-ALL cell line Jurkat, and healthy neutrophils cells.^39^ Due to differences between approaches including probe size, temperature and loading rate we cannot easily compare our results to those of other studies, nevertheless our results are within the same range (<1 kPa).^39^ We observed that THP-1 cells have a Young’s modulus of 113 Pa, which can be further increased upon retronectin treatment, in an ITGB1-dependent manner. This suggests that AML cells alter their biophysical properties upon interaction with the extracellular matrix. The BM niche is heterogeneous, implying that AML mechanical properties may be locally varied and accordingly impact processes such as migration.^29^^,^^45^^,^^58^^,^^59^ Also, we find that differences in elastic modulus exist between primary patient samples, which were not related to cell size. More likely, we propose that differences in intracellular signaling, instructed via the presence of specific mutations which differ considerably between patients, underlie such differences in biophysical properties, but further studies on larger cohorts of patient samples will be needed to uncover such relationships.

Cellular mechanical properties were previously linked to F-actin polymerization.^46^ When strong deformations were induced on the murine HSC-like cell line HPC-7 the cells became weaker and more deformable after pre-treatment with SDF1a, which binds to and activates the CXCR4 receptor.^46^ However, when smaller deformations were induced, the Young’s modulus increased, which was attributed by the authors to increased F-actin polymerization.^46^ In our study, when THP-1 cells were treated with retronectin to activate integrins ITGA4 and ITGA5, which both dimerize with ITGB1, we also observed differences in actin network organization. It is likely that the different Young’s modulus values measured by AFM for retronectin exposed WT and *ITGB1* KO cells are directly related to the differences in actin network organization as we expect indentations of ≥300 nm will have substantial contributions from the cell volume, in addition to the cortical stiffness that is probed at small indentations.^54^ In *ITGB1* KO cells, the actin network was mainly localized immediately under the plasma membrane, thus we expect that contributions from the actin enriched membrane region and the F-actin depleted volume resulted in an intermediate global cell elastic modulus. In retronectin exposed WT cells, the actin network is strongly present both at the cell membrane and throughout the cell volume and as such the global cell elastic modulus is higher than *ITGB1* KO cells. Besides a reduction in cytosolic actin filaments in *ITGB1* KO cells, we also noted an increase in actin-rich projections that resembled filopodia, and we hypothesize that these might play a role in processes such as cell sensing, migration, and adhesion to the extracellular matrix.^60^

Taken together, we have developed a platform that allows for reproducible AFM measurements to characterize their biophysical properties. Initially we measured the elastic properties of wild type and *ITGB1* knock out THP-1 cells in the presence of retronectin. *ITGB1-*retronectin binding results in changes in the actin cytoskeleton^27^ which is then anticipated to alter cellular mechanical properties. We show that activation of *ITGB1* does indeed lead to measurable changes in the Young’s modulus, likely due to cytoskeletal reorganization throughout the entire cell volume. We then utilized our approach to investigate the mechanical properties of primary AML patient samples. Our data indicate that clear differences exist in cell elastic modulus across patient samples, and that the most immature LSC populations also display a higher Young’s modulus compared to more committed leukemic cell populations. In certain cases, this might be linked to integrin-mediated actin cytoskeletal alterations. It will be interesting to determine in future studies how such biophysical properties are controlled by genetic alterations, and how these in turn impact on drug sensitivities.

### Limitations of the study

Here, we show a methodology to perform AFM indentations on non-adherent cells to determine their Young’s modulus. We then applied this to study the effect of *ITGB1* activation and stem cell maturity on the cellular elastic properties. Though our approach allows us to measure non-adherent cells while minimizing the effects of immobilization procedures on the cellular properties, there are some limitations.

Firstly, mechanical stimulation of the cells by the repeated indentations may induce changes in the cellular Young’s modulus. Though we show that the largest change in Young’s modulus occurs after the first indentation and we speculate that this is caused by repositioning of the cell in the microwell, we cannot rule out that the measured values may be influenced by repeated indentations. However, analyzing only the first indentation curves showed a similar trend (Figure S6) as the analysis for the remaining indentations. Thus, even if the magnitude of the measured Young’s moduli may be affected by mechanical stimulation, our conclusion that *ITGB1* activation by retronectin significantly impacts cellular biophysical properties remains consistent.

Similarly, some form of sample fixation is necessary for AFM measurement. Thus, we are not able to measure the non-adherent cells under truly free conditions. Using the loose confinement approach we describe here, we anticipate that the cells are much less perturbed compared to approaches where cells are adhered to the substrate.^45^^,^^46^^,^^47^^,^^48^ Nevertheless, we cannot rule out that the cellular biophysical properties are still somewhat altered by the confinement. However, we also note that, given the cell and PFPE well size, the majority of the cellular volume we probe is not in contact with the well walls. Moreover, the portion of the cell probed is exposed to the bulk solution. Hence we anticipate that any effect on the cells or their ability to interact with external stimuli due to the restriction of the walls contributes only to a minor extent.

Additionally, AFM is a low throughput technique. In the case of patient samples, this makes it challenging to capture the full heterogeneity of the cellular population and to obtain results for a large number of different patients. On the other hand, the single cell data obtained by AFM can in fact highlight the heterogeneity of the cell population which may be lost in higher throughput bulk techniques. Moreover, we show that even with limited numbers of cells we are able to observe statistically significant changes in the Young’s modulus of THP-1 cells which are in line with previous expectations, as well as in accordance with our confocal microscopy data. Likewise, we were able to observe differences in the immature and mature AML fractions of the patient samples. Nevertheless, we emphasize that our results for the patient AML samples give an early indication of potential population differences and that a larger patient cohort will be necessary to validate the universality of the findings. Moreover, investigating patients with different genetic mutations to those investigated here would be very interesting.

Lastly, in this study we only investigate the role of the ITGB1 subunit whereas there are a variety of relevant integrin subunits such as ITGA4, ITGA5, ITGA6, and ITGB3.^21^^,^^22^^,^^23^^,^^24^^,^^30^ ITGB1 was selected as it dimerizes with several of the alpha subunits to form integrins that are known to bind fibronectin. Thus, knock out of *ITGB1* served as a basis to determine whether differences in Young’s modulus could be measured by our approach for a system where cytoskeletal reorganization is well known.^25^^,^^26^^,^^27^ From this we were able to move to the clinically relevant case of patient AML samples. Nevertheless separate knock out of the aforementioned subunits could be very interesting to form a more detailed picture of the integrin mediated cellular response to retronectin and its effect on the Young’s modulus.

## Resource availability

### Lead contact

Requests for further information and resources should be directed to and will be fulfilled by the lead contact, Wouter H. Roos (w.h.roos@rug.nl).

### Materials availability

All unique/stable reagents generated in this study are available from the lead contact without restriction.

### Data and code availability

• All data reported in this paper will be shared by the lead contact upon request.
• This paper does not report original code.
• Any additional information required to reanalyze the data reported in this paper is available from the lead contact upon request.

## Acknowledgments

This work is supported by a Dieptestrategie grant of the Zernike Institute National Research Centre of the Rijksuniversiteit Groningen. We thank the Flow Cytometry Unit of the University Medical Center, Groningen. Moreover, we thank P. de Haan, and E. Verpoorte of the Pharmaceutical Analysis group, Groningen Research Institute of Pharmacy for the providing the photolithography mask used for the PFPE wells. We also thank G. Wlodarczyk-Biegun for providing the phalloidin.

## Author contributions

C.J.R. performed AFM and confocal experiments and analysis. A.T.J.W. performed the *ITGB1* knock-out and validation. A.Z.B.-V. defrosted and sorted the AML cells. E.K. performed and analyzed the adhesion experiments. L.S.D. performed preliminary AFM measurements. P.P.M.F.A.M. optimized and fabricated the PFPE wells. J.J.S., W.H.R., and G.P. designed and supervised the research. The manuscript was written with contributions from C.J.R., A.T.J.W., A.Z.B.-V., J.J.S., and W.H.R.

## Declaration of interests

There are no conflicts of interest to declare.

## STAR★Methods

### Key resources table

**Table:** 

REAGENT or RESOURCE	SOURCE	IDENTIFIER
**Antibodies**		

Mouse monoclonal anti-CD29 PE	Invitrogen	Cat# 12-0299-42; RRID: AB_2572555
Mouse monoclonal CD34-FITC	BD Biosciences	Cat#345801; RRID: AB_2868825
Mouse monoclonal CD38-PE	BD Biosciences	Cat#345806; RRID: RRID: AB_2868828
Mouse monoclonal CD45-PERCP	Biolegend	Cat#304026; RRID: AB_893341
Human monoclonal CD34-PE	Miltenyi Biotec	Cat#130-120-520; RRID: AB_2811343
Mouse monoclonal CD38-FITC BD	BD Pharmingen	Cat#555459; RRID: AB_395852
Mouse monoclonal CD45-PE-Cy7	Biolegend	Cat#368532; RRID: AB_2715891

**Biological samples**		

Bone marrow or peripheral blood samples of AML patients	University Medical Center Groningen	This paper

**Chemicals, peptides, and recombinant proteins**		

Retronectin	Takara Bio	Cat#T100B
TrypLE Express	Gibco	Cat #12604021
SU-8 2002	micro resist technology GmbH	N/A
Sylgard 184 Polydimethylsiloxane	Mavom	Cat#1060040
Fluorolink MD 700	Acota Ltd.	N/A
2-Hydroxy-2-methylpropiophenone	Sigma Aldrich	Cat#405655
Alexa Fluor® 488 phalloidin	Cell Signaling Technology	Cat#8878

**Experimental models: Cell lines**		

Human: THP-1	DSMZ	ACC16

**Oligonucleotides**		

Single guide RNA, DNA template sequence: GATCATTTAGGTGACACTATAGTGGAGAATGTATACAAGCA GTTTAAGAGCTATGCTGGAAACAGCATAGCAAGTTTAAATA AGGCTAGTCCGTTATCAACTTGAAAAAGTGGC ACCGAGTCGGTGC	Eurofins genomics, Ebersberg, Germany	N/A
Primer exon3_for: GATAAAGGCCTGAGGGGATG	Eurofins genomics, Ebersberg, Germany	N/A
Primer exon3_rev: GTGCTCAATAGCCACATCTG	Eurofins genomics, Ebersberg, Germany	N/A

**Software and algorithms**		

ImageJ/FIJI	Schneider et al.,^61^ Schindelin et al.^62^	https://imagej.nih.gov/ij/
FlowJo	FlowJo, LLC	https://www.flowjo.com/
JPK SPM Data Processing	Bruker	N/A

**Other**		

Duke Standards™ 20 μm borosilicate glass microspheres	Thermo Fisher Scientific	Cat#9020
CB3 cantilever of the qp-BioAC-50 chip	NANOSENSORS	qp-BioAC-50

### Experimental model and study participant details

#### THP-1 cell culture and knock-out

*ITGB1* was knocked out in THP-1 cells (DSMZ, ACC-16, sex: male), essentially according to the method described in reference [55], with minor modifications. Three single guide RNAs (sgRNA) were designed using the online platform Benchling (www.benchling.com), of which the best performing was chosen (sequence: GTGGAGAATGTATACAAGCA) based on initial experiments. One million THP-1 cells were electroporated with 20 μg Cas9 complexed with 12 μg *in vitro* transcribed sgRNA using an Amaxa Nucleofector I device (Lonza) using program D-06 after which the cells were cultured in 4 ml of growth medium consisting of RPMI medium (Gibco) supplemented with 10% fetal calf serum (Sigma-Aldrich) for 5 days at 37°C, 5% CO_2_ and a humidified atmosphere. Cells were stained with anti-CD29 PE (cat#12-0299-42, Invitrogen) and negative cells were sorted using a MoFlo XDP cell sorter (Beckman Coulter). Expanded cells were analysed for frameshift mutations causing a preliminary stop codon by Sanger sequencing using the primers: exon3_for: GATAAAGGCCTGAGGGGATG and exon3_rev: GTGCTCAATAGCCACATCTG. Tide analysis (www.tide.nki.nl) revealed that 97% of the cells contained a stop codon inducing mutation. Re-analysis by flowcytometry showed that 98% of the cells were ITGB1 negative (Figure S2). The cells were expanded, tested negative for mycoplasma and either frozen or cultured for further use.

#### AML cell isolation and *in vitro* cell culture

Bone marrow or peripheral blood samples of AML patients were studied after informed consent and protocol approval by the Ethical Committee in accordance with the Declaration of Helsinki. Mononuclear cells (MNCs) were isolated via Lymphoprep (cat#31858-6, Progen ) density gradient-based separation and cryopreserved.

Cryopreserved AML patient samples were thawed and resuspended in newborn calf serum (NCS) supplemented with DNase I (20 Units/mL), 4 mM MgSO_4_ and heparin (5 Units/mL) and incubated at 37°C for 15 min. AML MNCs were blocked for 5 minutes with human FcR blocking reagent (Miltenyi Biotec) and stained with antibodies: (CD34-FITC cat#345801, BD Biosciences (2 μl/100 μl); CD38-PE cat#345806, BD Biosciences (3 μl/100 μl); and CD45-PERCP cat#304026, Biolegend (1.5 μl/100 μl)) or with antibodies: (CD34-PE cat#130-120-520, Miltenyi Biotec (2μl /100 μl); CD38-FITC cat#555459, BD Pharmingen (3μl/100μl); and CD45-PE-Cy7 cat#368532, Biolegend (1.5 μl/100 μl)). Viability was assessed by DAPI staining. CD34^+^/CD38^-^ (defined as the 10% lowest CD38) and CD34^+^/CD38^+^ (defined as 30% highest CD38) cells were sorted on the Moflow Astrios (Beckman Coulter).

Sorted cells were cultured in liquid culture medium consisting of αMEM (Gibco) supplemented with 25% fetal calf serum, 1% penicillin and streptomycin (Life Technologies, Grand Island, USA), with the addition of 20 ng/mL G-CSF (Amgen), N-Plate (clinical grade TPO) (Amgen) IL-3 (Sandoz), 500 nM SR1 (cat#182706, Calbiochem) and 35 nM UM171 (Bocsci). Liquid cultures were grown at 37°C and 5% CO_2_ in a humidified atmosphere. Cells were measured with AFM indentation one or two days after defrosting. To prevent biases from the time in culture, the CD34^+^/CD38^+^ cells were measured before the CD34^+^/CD38^-^ cells for patients 1 and 3, whereas the order was reversed for patient 2.

### Method details

#### THP-1 cell controls

Cell proliferation was measured by loading 20 μl of cell suspension into a Neubauer hemacytometer and the cells within 8 quadrants were manually counted. This was repeated each 24 h. For the cell adhesion experiments, 12 well culture plates were coated with retronectin (cat#T100B, Takara Bio) according to the manufacturer’s recommendations; overnight at 4°C followed by a blocking step using 2% BSA/PBS solution (Roche). THP-1 cells were plated on the coated plates and incubated at 37°C for 2 hours. Medium including non-adherent cells was pipetted off and collected, repeated by two PBS washing steps to collect all non-adherent cells. Adherent cells were dissociated using TrypLE Express (Gibco) and collected by PBS washes. Both non-adherent and adherent cells were counted using the CytoFLEX flow cytometer (Bechman Coulter Brea) and analyzed by FlowJo software.

#### PFPE wells

A photolithography approach and multiple molding steps with elastomer were performed to produce PFPE chips with a grid of wells.^50^^,^^51^^,^^63^^,^^64^ First, a SU-8 2002 (micro resist technology GmbH) master mold was fabricated with ∼4 μm high and 10 μm diameter round protrusions with a 25 μm pitch, followed by preparation of a polydimethylsiloxane (PDMS, Sylgard 184) elastomer mold of the master as described previously in references [48, 49]. PDMS was then cast into the PDMS mold and cured at 70°C for 70 min to produce a negative version of the final structure. Lastly, perfluoropolyether (PFPE) was prepared from Fluorolink MD 700 (Acota Ltd.) and 2% photointiator 2-Hydroxy-2-methylpropiophenone (Sigma Aldrich). The PFPE mixture was placed into the negative PDMS molds and placed under a nitrogen stream for 10 min. The PFPE mixture was cured in a BlueWave LED flood-curing system (Dymax Europe GmbH) at 3 J/cm^2^. The PFPE wells were removed from the PDMS molds for further use. PFPE wells were imaged by atomic force microscopy using a JPK NanoWizard (Bruker) mounted on an optical microscope (Olympus) in air using quantitative imaging (QI) mode. Cantilever A of the NITRA-TALL-R-G chip (AppNano) with a nominal spring constant of 0.1 N/m and tip length of 14-16 μm was used to image the wells. Cantilevers were calibrated using contact-based calibration. Analysis of the AFM images was performed in the JPK SPM Data Processing software. The PFPE substrate was also measured following the same procedure as performed on cells (see below for details).

#### Colloidal probe cantilevers

Duke Standards™ 20 μm borosilicate glass microspheres (Thermo Fisher Scientific) were glued to the CB3 cantilever of the qp-BioAC-50 chip (NANOSENSORS) with a nominal spring constant of 0.06 N/m. The glass beads were streaked onto a glass slide and a streak of two component epoxy glue (Bison) was placed in the vicinity of the beads. Using a MultiMode Nanoscope V (Bruker) the cantilever was pressed into the glue, retracted, moved over the center of a singular glass bead, and then lowered onto the bead to adhere it to the cantilever.^65^ Colloidal probe cantilevers were left to cure for at least two days before use.

#### Atomic force microscopy indentation

Cell indentation was performed on a JPK NanoWizard mounted on an optical microscope. PFPE wells were placed in the BioCell holder (JPK) and preincubated with phosphate buffered saline (PBS, Gibco) for 1 h at 37°C, with the colloidal probe cantilever in the liquid. 50% of the PBS was removed and replaced with cells in medium, therefore the serum concentration for the experiments was half of that used for the culture conditions. For retronectin exposed samples, retronectin was pre-mixed with the cells before adding to the BioCell to achieve a final retronectin concentration of 1 μg/ml. Cells were then incubated in the BioCell for 1 h at 37°C with the cantilever in the liquid to allow cells to settle and for the system to equilibrate. Prior to indentation, the colloidal probe cantilevers were calibrated using the contact free approach. The cantilever was placed with the bead centre over the centre of a cell located within one of the PFPE wells. 15 force-distance curves were then performed on each cell with a setpoint of 1 nN, extend speed of 1 μm/s, and piezo z-length of 3.5 μm. The majority of cells were indented between 300 nm and 2.5 μm before reaching the 1 nN setpoint, *i.e.* no more than ∼20% of the cell diameter. We do note that it has been reported that substrate effects can influence the Young’s modulus obtained from the Hertzian fit for indentations greater than 10% of the object diameter.^66^ However, in the cases where cells were indented more than 10% we did not observe obvious deviations from the Hertz fit for the different segments across the same force curves (*i.e.* the slope up to 1 μm depth was similar to the slope from 1 – 2.5 μm depth). From this we conclude that substrate effects were negligible.^39^ All measurements were taken within 4 h of placing cells on the microscope to limit effects of decreased cell viability under ambient CO_2_ conditions and sample evaporation.

Before indentation, an image of the cell to be measured was taken with the optical microscope. Cell areas were then manually determined from the optical images using ImageJ/Fiji software.^61^^,^^62^ Analysis of the force-distance curves was performed using JPK SPM Data Processing software. The curves from individual cells were batch processed using the software in-built Hertz-fit process. In short, the force distance curves were automatically corrected for the baseline offset and contact point offset, and then the vertical tip position was corrected for the bending of the cantilever. The corrected approach curves were then fit using the Hertz/Sneddon model with a spherical probe diameter of 20 μm and Poisson ratio of 0.5.

#### Confocal microscopy

Glass slides (#1.5, Marienfeld) were incubated with 50 μg/ml retronectin for 4 h at room temperature and washed with PBS. THP-1 WT or *ITGB1* KO cells were then seeded onto the retronectin coated glass slides and left overnight to adhere. Retronectin was added to the cell medium to achieve a final concentration of 1 μg/ml and incubated for 1 h. Cells were then washed with PBS and fixed with 4% paraformaldehyde (VWR) for 15 min. Cells were washed with PBS and then permeabilized using 0.1% triton-X-100 (Sigma Aldrich) for 5 min. Cells were washed again and then incubated with 0.33 μM solution of Alexa Fluor® 488 phalloidin (Cell Signaling Technology) for 15 min followed by washing. Cells were then imaged with a Zeiss LSM 710 confocal microscope (Zeiss) using the 488 nm excitation laser.

To quantify the differences in actin network between the two cell conditions, the higher resolution images of individual cells, such as those presented in Figures 3B, 3D, 3G, and 3I were analysed. A single optical section from the midpoint of each cells was used. Background subtraction was performed in ImageJ/Fiji^61^^,^^62^ using the rolling ball background removal with a radius of 20 pixels. Images were then smoothed to reduce the noise introduced by the background subtraction. The images were then thresholded to produce a binary image of the actin structures, which was then skeletonized.^37^ The Analyze Skeleton tool was used to yield skeleton lengths.

### Quantification and statistical analysis

Experiments using THP-1 cells were performed in triplicate. Experiments using patient samples were performed once for each patient. The number of cells, *n*, and results of the significance testing for Figure 2 can be found in the captions. For Figure 4 they are reported in Tables 2 and S4. The number of cells, *n*, for Tables 1 and 2 are reported in the tables themselves. Where applicable, the mean and error are defined in the captions of all main and supplemental figures and tables.

#### THP-1 cell controls

For the proliferation experiments, each cell count value was normalized by dividing it by the mean count of the 8 quadrants from the first day. The reported values are the mean and standard deviation of the normalized counts across the 8 quadrants for each day. For the cell adhesion experiments the reported values are the mean and standard deviation across the three repeat experiments. Significance testing was performed with a two sample t-test with an alpha of 0.05.

#### Atomic force microscopy indentation

Cells that showed major morphological changes or signs of cell death after indentation based on optical inspection were removed from the dataset, however this occurred rarely: 4 cells compared to the final number of 461 cells presented in Figures 2 and 4 (including the outlier cells). Likewise, cells that moved out of the wells during repeated indentation were also removed from the dataset (31 cells moved compared to the final 461 cells). The fit and quality of each force curve was manually checked. Force curves that did not have a flat baseline of at least 500 nm were removed from the data set. Likewise, if the indentation showed features such as membrane-pinching events, then these force-distance curves were also discarded. Cells with less than 11 force curves remaining after quality assessment were not included in the dataset. For each of the remaining cells, the first indentation was removed and then the Young’s modulus was averaged across the remaining force curves per cell (≥ 10 curves).

Outliers were identified as lying beyond the population first and third quartiles by more than 1.5 times the interquartile range. This approach was chosen as it is a non-parametric method which makes no assumptions about the shape of the data. To validate the outlier analysis a post hoc z test was used. All identified outliers had z scores ≥ 3 and thus deviated significantly from the mean. The identified outliers were then removed from the dataset to determine the population statistics reported in Tables 1 and 2. The average Young’s modulus value per cell was averaged across all cells within the group to obtain the population mean. The reported errors are the standard errors and *n* is the number of cells included in the dataset of each population. Significance testing was performed with a two sample t-test with an alpha of 0.05. Less stringent outlier analysis was performed by increasing the threshold to 3 times the interquartile range.

#### Confocal microscopy

The cortical actin often produced one or two very long skeletons due to the high intensity and density of staining in that region. Therefore, the skeletons associated with the cortex were removed from the dataset. Moreover any skeletons with a length less than 250 nm were also removed as these are below the resolution limit.^37^ The quoted values for the total actin length and average actin length are the mean across cells whilst the errors are the standard error of the mean across cells. For the WT and *ITGB1* cells exposed to retronectin a total of 22 and 20 cells were measured, respectively, from images from three replicates.
