# Supplementary material for: Elastic properties of leukemic cells linked to maturation stage and integrin activation
Source: iScience. 2025 Mar 4;28(4):112150. doi: 10.1016/j.isci.2025.112150 (PMC11978321; doi:10.1016/j.isci.2025.112150)
Supplement: Document S1. Figures S1–S8 and Tables S1–S4 [file mmc1.pdf]

iScience, Volume *28*

Supplemental information

Elastic properties of leukemic cells

linked to maturation stage

and integrin activation

Ceri J. Richards, Albertus T.J. Wierenga, Annet Z. Brouwers-Vos, Emmanouil Kyrloglou, Laura S. Dillingh, Patty P.M.F.A. Mulder, Georgios Palasantzas, Jan Jacob Schuringa, and Wouter H. Roos

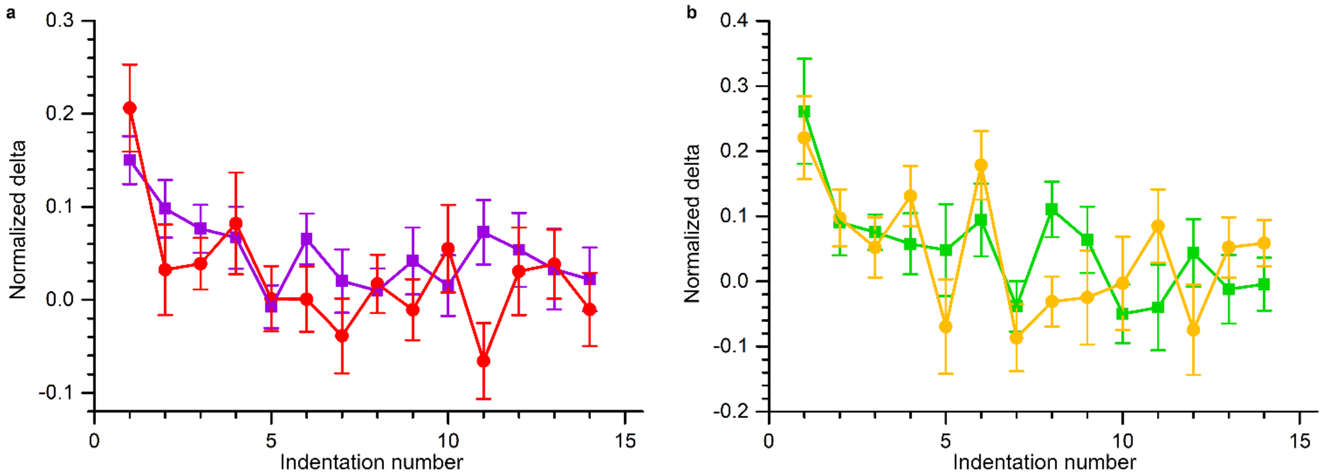

Figure S1 Measured Young's modulus changes with successive indentations. Related to Figure 2. The Young's modulus of THP-1 cells measured using AFM were averaged across cells for each individual indentation. The normalized change in Young's modulus, delta, was calculated as $\frac{E_{n+1}-E_n}{E_1}$, where E_n is the average Young's modulus value of indentation number *n*. Normalized delta values for **(a)** THP-1 wild type cells under control conditions (purple squares) and in the presence of retronectin (red circles), and **(b)** THP-1 *ITGB1* knock out cells under control conditions (green squares) and in the presence of retronectin (yellow circles). Error bars are the standard errors. The highest delta values are observed between the first and second indentation for all conditions, after which the delta values are lower and sometimes negative (*i.e.* an decrease in average Young's modulus with indentation number). Thus the greatest change in Young's modulus occurs after the first indentation which we hypothesize is caused by repositioning of the cell within the well.

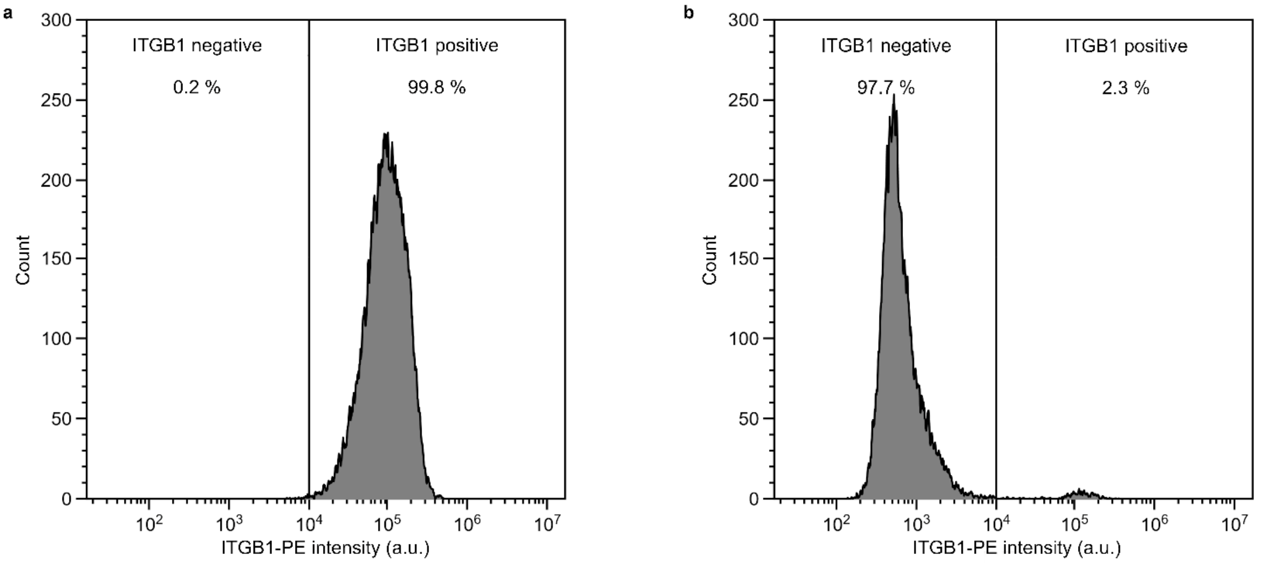

Figure S2. Validation of *ITGB1* knock out in THP-1 cells. Related to STAR Methods. THP-1 cells were stained for ITGB1 and then cell fluorescence intensities in the PE channel were measured with flow cytometry for **(a)** wild type, and **(b)** *ITGB1* knock out THP-1 cells. Increased PE signal indicates increased presence of ITGB1. The wild type cells showed high PE intensities, which was used to bench mark the gates for identifying ITGB1 positive and negative cells (black vertical line). 98% of *ITGB1* knock out THP-1 cells had PE intensities lower than the threshold (negative for ITGB1) whilst a small population had higher intensities (positive for ITGB1).

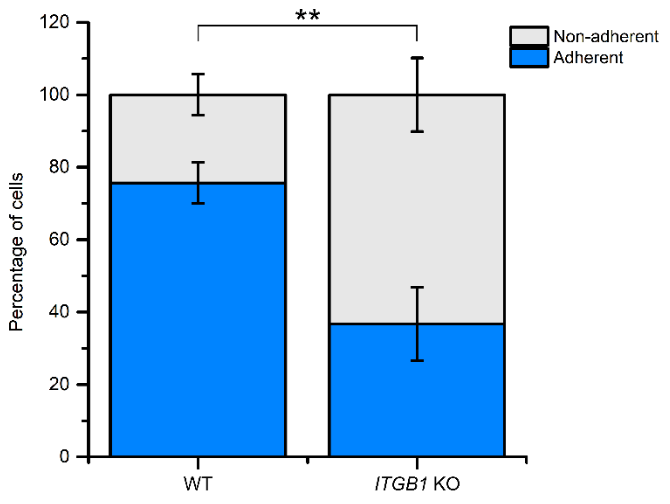

Figure S3. Wild type and *ITGB1* knock out THP-1 cell adhesion. Related to STAR Methods. Cells were seeded onto retronectin coated plates and the adherent (blue) and non-adherent (grey) fractions were measured by flow cytometry. The percentage of WT adherent cells significantly differed from the percentage of *ITGB1* KO adherent cells (p=0.009, two sample t test). Thus, the *ITGB1* KO resulted in reduced cellular adhesion to retronectin. Values shown are the mean and standard deviation across triplicate experiments. The total number of counted cells, *n*, for each repeat was 58219, 59148, and 63817 for the WT THP-1 cells, and 64064, 68871, and 62225 for the *ITGB1* KO cells.

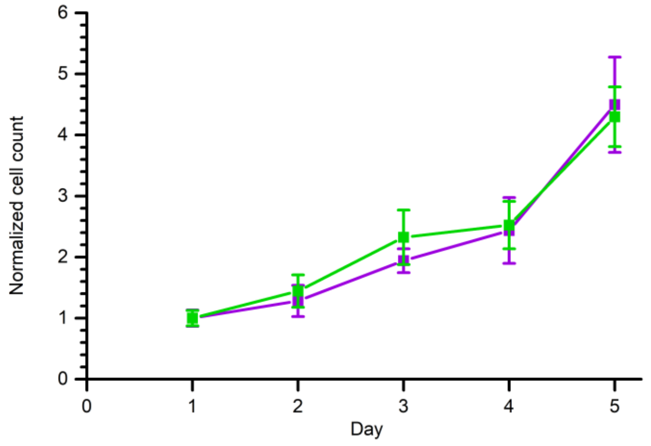

Figure S4. Proliferation rates of wild type and *ITGB1* knock out THP-1 cells. Related to STAR Methods. Cells were counted each 24 h for wild type (purple) and *ITGB1* knock out (green) THP-1 cells. The counts were normalized to the average count of the first day. Both wild type and knock out cells show similar proliferation rates across 5 days with a doubling time of approximately 2 days. Values are the mean and standard deviation across counts from 8 quadrants.

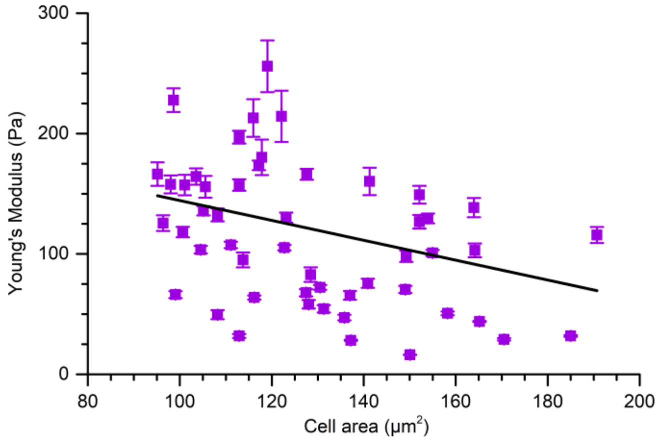

Figure S5. Cell Young's modulus as a function of cell area. Related to Figure 2. Plot of individual cell Young's moduli as a function of cell area for THP-1 wild type cells under control conditions. Data are the average Young's modulus values over 10-14 indentations per cell (first indentations were removed) and the error bars are the standard errors. The linear fit to the data (black line) suggests there is only a weak negative correlation between Young's modulus and cell area (Pearson's r of -0.25)

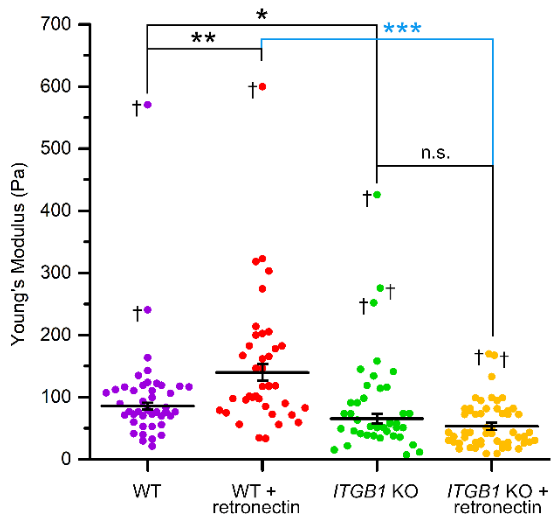

Figure S6. Young's modulus values of THP-1 cells obtained from the first indentation curves only. Related to Figure 2. Distribution of the measured Young's modulus values for cells under various conditions. Each point is the value obtained from the first indentation performed on an individual cell. All experiments were performed in triplicate and outliers are indicated with the daggers. The horizontal bars show the distribution means and the error bars are the standard error across cells after outlier removal. The following means and standard errors were obtained: 86 ± 5 Pa, 140 ± 13 Pa, 67 ± 7 Pa, and 53 ± 6 Pa for WT, WT+ retronectin, *ITGB1* KO, and *ITGB1* KO + retronectin, respectively. Significance testing was performed using a two sample t-test with an alpha of 0.05. The following p values were obtained: p = 0.001, 0.017, 0.636, and 4 × 10^{-6} for WT/WT+ retronectin, WT/*ITGB1* KO, *ITGB1* KO/*ITGB1* KO + retronectin, and WT+ retronectin/*ITGB1* KO + retronectin, respectively. Analysing only the first indentation curves results in a lower Young's modulus value for each condition compared to analysing curves 2-15 (Table 1). However, the trends remain similar, *i.e. w*ith this analysis WT cells still show a significant increase in Young's modulus when exposed to retronectin compared to control conditions, where *ITGB1* KO show no significant changes due to retronectin exposure. Unlike the results presented in Figure 2e, there is a small significant difference in the Young's modulus values of the WT and *ITGB1* KO cells under control conditions.

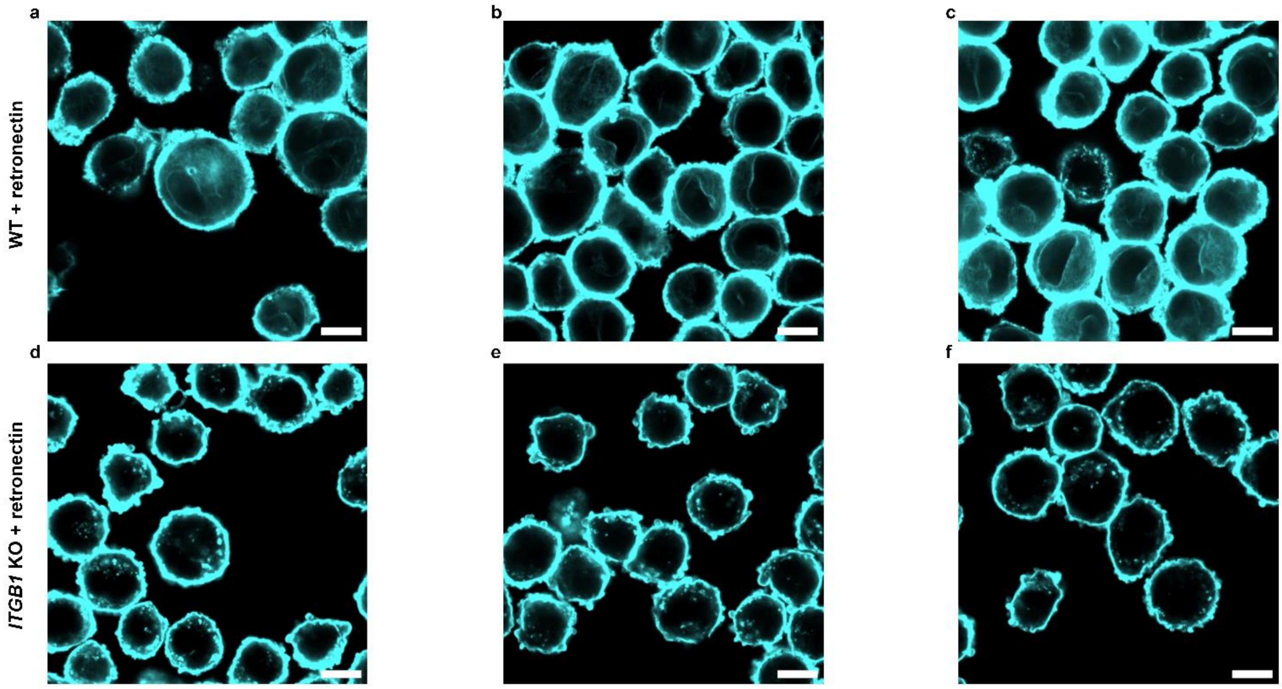

Figure S7. Additional images of actin morphology in retronectin exposed THP-1 cells. Related to Figure 3. Additional overview confocal images of **(a–c)** wild type and **(d–f)** *ITGB1* knock out THP-1 cells exposed to retronectin. THP-1 cells were seeded on retronectin coated slides and then exposed to additional retronectin in solution. Actin filaments were stained using phalloidin (cyan) and imaged with confocal microscopy. The morphologies are similar to those displayed in Figure 3, *i.e.* WT cells show extended cytoplasmic staining whereas *ITGB1* knock out cells show distinct puncta. Scale bars are 10 µm.

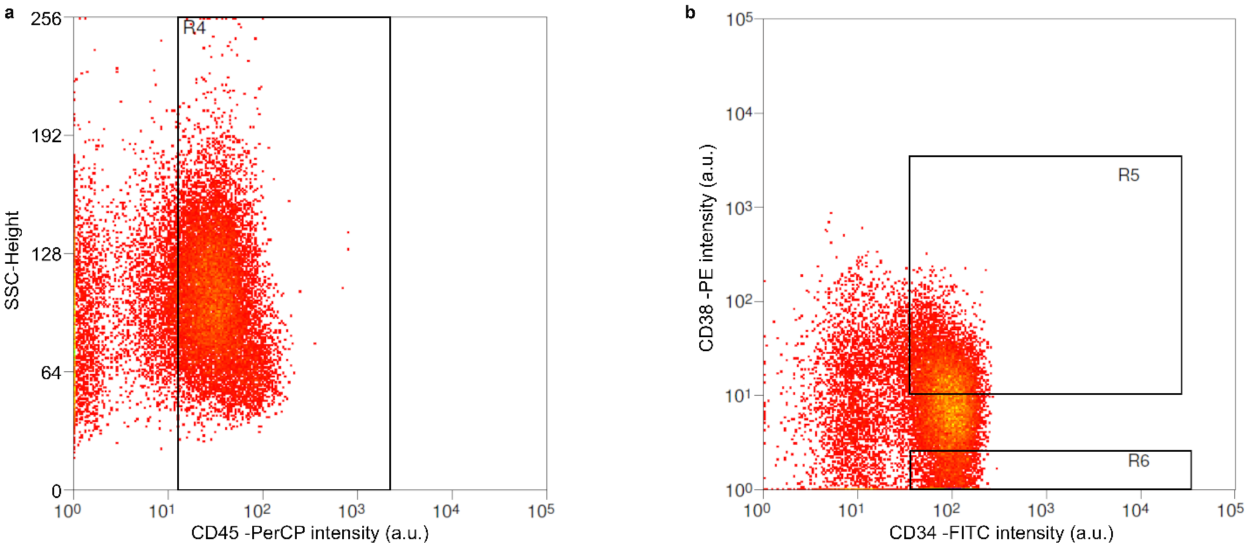

Figure S8. Patient derived AML cell sorts. Related to STAR Methods. Patient samples were initially sorted for viable single cells using flow assisted cells sorting (FACS). **(a)** The population was then sorted for the blast cell marker CD45. The side scattering channel (SSC) was compared to the PerCP channel, where higher PerCP intensities indicate cells that were positive for CD45 (region 4). **(b)** The cells were then subsequently sorted for the leukemic cell marker CD34 and progenitor cell marker CD38. The $CD34^{+}/CD38^{+}$ cell population was sorted by taking the top 30% of cells in the CD38 channel that were also positive for CD34 (region 5), and $CD34^{+}/CD38^{-}$ were likewise sorted by taking the bottom 10% (region 6). The data displayed are for the sample from patient 2. The number of sorted cells is reported in Table S1.

Table S1 Numbers of cells in the sorted populations. Related to Figure 4. The number of cells in the pre-sorted population and in the sorted $CD34^+/CD38^+$ and $CD34^+/CD38^-$ populations used for AFM experiments. Here pre-sorted refers to the viable population of singlet cells. After sorting, cells were centrifuged, resuspended and left in culture at least overnight before measurement. Thus, it is likely that the ultimate samples contained fewer cells than quoted here. We also note that half of each population was used per AFM measurement to investigate both the conditions with and without retronectin.

Sort	Number of cells		
	**Patient 1**	**Patient 2**	**Patient 3**
**Pre-sorted**	19539	19323	19701
**$CD34^+/CD38^+$**	3920	5829	5472
**$CD34^+/CD38^-$**	1225	2820	1930

Table S2 Patient mutations. Related to Figure 4. Genetic mutations present in the patient AML samples. The three patient samples were chosen to have similar genetic backgrounds in terms of NPM1 and FLT3. (ND=not determined, VAFs of mutations are indicated between brackets).

AML sample	NPM1	FLT3	Karyotype	Additional mutations
**Patient 1**	wt	wt	-5	ND
**Patient 2**	wt	wt	complex	ND
**Patient 3**	wt	wt	45, XY, -7, del(12)(p11p12)[7]/46, XY[3]	DNMT3A (0,54); SRSF2 (0,5); SETBP1 (0,42); PTPN11 (0,1); NRAS (0,09); KRAS (0,06)

Table S3 Cell area measurements of patient samples. Related to Figure 4. Cell areas were determined from optical microscopy and the values stated are averages and standard deviations.

Sample	Cell Area (μm^2)		
	**Patient 1**	**Patient 2**	**Patient 3**
**$CD34^+/CD38^+$**	92 ± 18	72 ± 20	64 ± 14
**$CD34^+/CD38^+$ + retronectin**	86 ± 18	72 ± 20	67 ± 11
**$CD34^+/CD38^-$**	71 ± 22	63 ± 10	67 ± 21
**$CD34^+/CD38^-$ + retronectin**	61 ± 16	55 ± 10	72 ± 17

Table S4 Statistical analysis of patient samples. *p* values obtained from a two sample t-test of the Young's modulus values obtained for each cell population. * indicates when two sample populations where found to have significantly different means using an alpha of 0.05.

**AML sample**	**$CD34^+/CD38^+$ + retronectin**	**$CD34^+/CD38^-$**	**$CD34^+/CD38^-$ + retronectin**
**$CD34^+/CD38^+$**	Patient 1: p = 0.106<br>Patient 2: p = 0.958<br>Patient 3: p = 0.386	Patient 1: p = 0.019 *<br>Patient 2: p = 0.0006 *<br>Patient 3: p = 0.008 *	---
**$CD34^+/CD38^+$ + retronectin**	---	---	Patient 1: p = 0.0001 *<br>Patient 2: p = 0.189<br>Patient 3: p = 0.241
**$CD34^+/CD38^-$**	---	---	Patient 1: p = 0.084<br>Patient 2: p = 0.082<br>Patient 3: p = 0.008 *
